# Myeloid-derived suppressor cells: an emerging target for anticancer immunotherapy

**DOI:** 10.1186/s12943-022-01657-y

**Published:** 2022-09-26

**Authors:** Yuze Wu, Ming Yi, Mengke Niu, Qi Mei, Kongming Wu

**Affiliations:** 1 Department of Oncology, Tongji Hospital of Tongji Medical College, Huazhong University of Science and Technology, 1095 Jiefang Avenue, Wuhan, 430030 People’s Republic of China; 2 Department of Breast Surgery, Zhejiang University School of Medicine First Affiliated Hospital, Hangzhou, 310003 China; 3 Cancer Center, Shanxi Bethune Hospital, Shanxi Academy of Medical Science, Tongji Shanxi Hospital, Third Hospital of Shanxi Medical University, Taiyuan, Shanxi People’s Republic of China

**Keywords:** Myeloid-derived suppressor cells, Cancer immunotherapy, Immune checkpoint inhibitors, The tumor microenvironment, Arginase I, Inducible nitric oxide synthase

## Abstract

The clinical responses observed following treatment with immune checkpoint inhibitors (ICIs) support immunotherapy as a potential anticancer treatment. However, a large proportion of patients cannot benefit from it due to resistance or relapse, which is most likely attributable to the multiple immunosuppressive cells in the tumor microenvironment (TME). Myeloid-derived suppressor cells (MDSCs), a heterogeneous array of pathologically activated immature cells, are a chief component of immunosuppressive networks. These cells potently suppress T-cell activity and thus contribute to the immune escape of malignant tumors. New findings indicate that targeting MDSCs might be an alternative and promising target for immunotherapy, reshaping the immunosuppressive microenvironment and enhancing the efficacy of cancer immunotherapy. In this review, we focus primarily on the classification and inhibitory function of MDSCs and the crosstalk between MDSCs and other myeloid cells. We also briefly summarize the latest approaches to therapies targeting MDSCs.

## Background

The tumor microenvironment (TME) releases multiple soluble factors that mediate normal myeloid differentiation and convert myeloid cells into immunosuppressive cells. This creates a tumor-promoting ‘macroenvironment’, which substantially limits the efficacy of cancer immunotherapy [1]. MDSCs are a cluster of cells with potent immunosuppressive effects widely distributed in the spleen and tumor tissues of tumor-bearing mice or the peripheral blood and tumor sites of cancer patients [2]. Under normal physiology, bone marrow cells differentiate from multipotent hematopoietic stem cells (HSCs) into diverse mature subsets, and macrophages, dendritic cells (DCs), and granulocytes are the terminally differentiated cells [3]. In contrast, in cancer conditions, the tumor microenvironment renders MDSCs incapable of differentiation, resulting in a population of immature heterogeneous cells [4]. Recent studies have increasingly emphasized that high concentrations of MDSCs are dramatically related to poor prognosis, cancer development and responses to immunotherapies in patients with breast, colorectal, and lung cancers and hematologic malignancies [5–8]. In the next section, we specifically discuss the classification and suppressive mechanisms of MDSCs. In addition, we emphasize the sophisticated crosstalk of MDSCs with bone marrow-derived cells and present clinically promising therapies targeting MDSCs.

## Phenotypes and classifications of MDSCs

As early as 1978, it was found in tumor-bearing mice that coculture of activated T cells with bone marrow cells suppressed T cells [9]. Later, this group of cells with immunosuppressive function, which accumulated significantly in the peripheral blood of cancer patients, was renamed immature myeloid cells (IMCs) and myeloid suppressor cells (MSCs ) [10, 11]. To avoid confusion, Gabrilovich et al. proposed the term MDSCs, which more precisely reflects the origin and function of these cells [12]. First, they were characterized in mice by the coexpression of CD11b and Gr 1 [13]. Then, based on the different expression levels of Ly6G and Ly6C, two different epitopes binding to Gr1, MDSCs were identified as two distinguished subsets: polymorphonuclear- (PMN-) and monocytic- (M-) MDSCs [14].

**Fig. 1:**
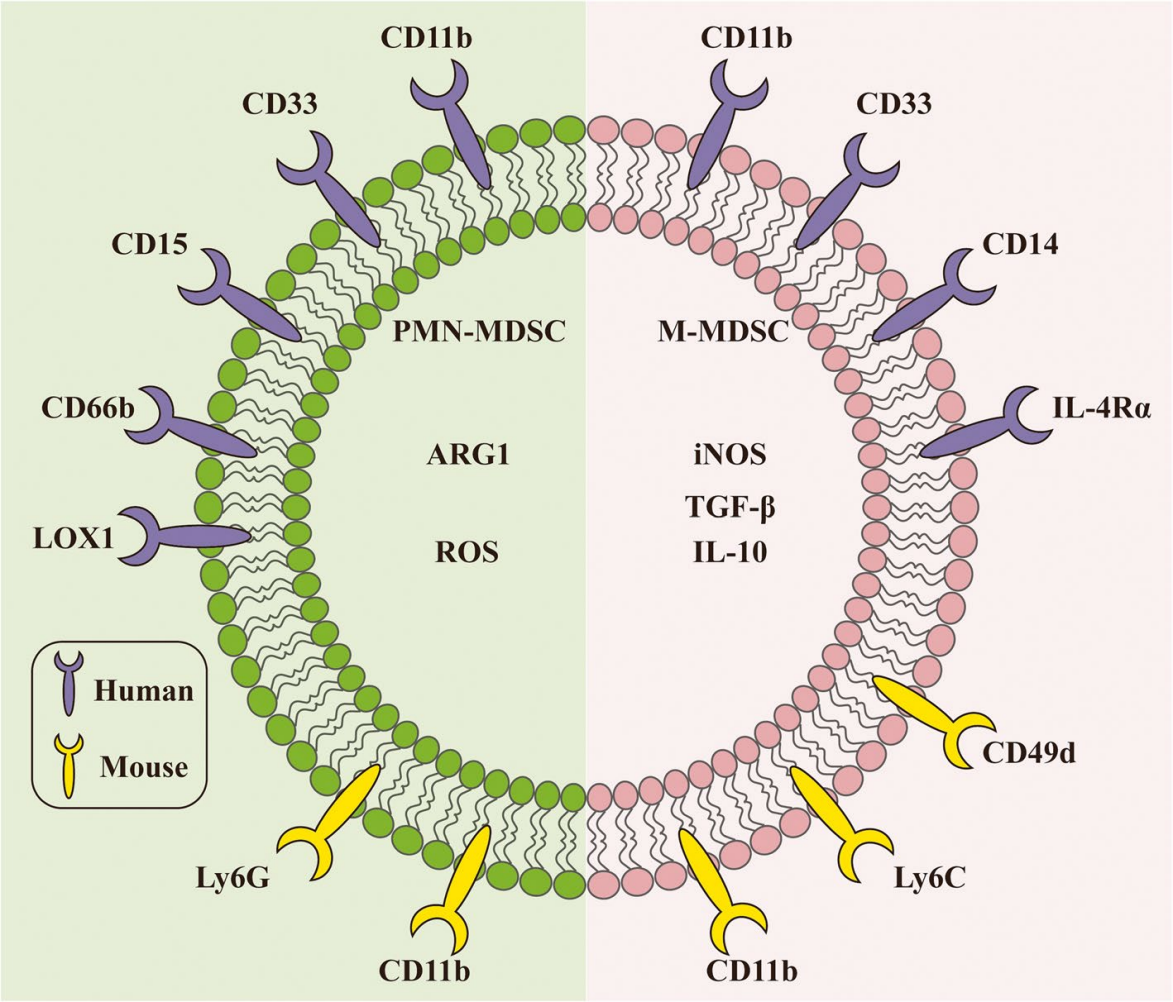
The phenotypes of PMN-MDSCs and M-MDSCs. In mice, PMN-MDSCs are defined as CD11b^+^Ly6G^+^Ly6C^lo^, and M-MDSCs are defined as CD11b^+^Ly6G^−^Ly6C^hi^. Human PMN-MDSCs are defined as CD11b ^+^ CD14^−^ CD15^+^ or CD11b^+^ CD14 ^-^ CD66b^+^, and human M-MDSCs are defined as CD11b^+^ CD14^+^ HLA-DR^−/low^ CD15^−^. In addition, several other markers have been associated with the MDSCs phenotype, such as CD49d, LOX1 and IL-4Rα

In mice, PMN-MDSCs are defined as CD11b^+^Ly6G^+^Ly6C^lo^, and M-MDSCs are defined as CD11b^+^Ly6G^−^Ly6C^hi^. Intriguingly, a recent study identified a new group of monocyte lineage precursors that differentiated into a substantial subset of PMN-MDSCs, and they were designated as monocyte-like precursors of granulocytes (MLPGs) [15]. In addition, several other markers have been associated with the MDSCs phenotype (Fig. 1). CD49d, a member of the integrin protein family, is only detected on M-MDSCs, not PMN-MDSCs. Haile et al. proposed that CD49d could substitute for Gr1 and, together with CD11b, better classify MDSCs [16].

MDSCs then received increasing attention in clinical practice. A number of studies have demonstrated that increased levels of MDSCs positively correlated with poor prognosis and clinical stage in patients with breast cancer, hepatocellular carcinoma, thyroid carcinoma, and non-small cell lung carcinoma (NSCLC ) [17–20]. MDSCs are of great value in predicting therapeutic effects in multiple solid tumors [21–23]. Human MDSCs lack typical markers of mature immune cells (Lin^−^, HLA-DR^−^) but express CD33, CD34, CD11b and IL-4Rα (CD124) [24, 25]. However, because human cells do not express Gr1, the phenotypes of human MDSCs remain controversial. Currently, human PMN-MDSCs are defined as CD11b ^+^ CD14^−^ CD15^+^ CD66b^+^ and human M-MDSCs as CD11b^+^ CD14^+^ HLA-DR^−/low^ CD15 − [14].

The majority of MDSCs are PMN-MDSCs, accounting for more than 75%, with M-MDSCs accounting for only 10–20 % [26]. Youn et al. examined the shared mechanism of MDSCs amplification in 10 diverse tumor models. Preferential expansion of PMN-MDSCs was shown in almost all tumor models, though the extent of amplification differed [27]. It is worth noting that M-MDSCs have a greater capacity for immunosuppression than PMN-MDSCs [27–29]. PMN-MDSCs preferentially use reactive oxygen species (ROS) and arginase I (ARG1) to mediate immunosuppression and are independent of inducible nitric oxide synthase (iNOS) [30], while M-MDSC-mediated inhibition mostly relies on nitric oxide (NO) and the suppressive cytokines IL-10 and TGF-β [4, 26] (Fig. 1). Importantly, MDSCs differentially drive immune suppression in a sex-specific manner. Male mice possessed elevated M-MDSCs in the tumor tissues, while females exhibited enhanced PMN-MDSCs in the peripheral circulation [31].

MDSCs phenotypes are similar to those of neutrophils and monocytes, making it a priority to identify MDSCs from other myeloid cells in peripheral blood **(**Table 1). PMN-MDSCs and neutrophils share mostly identical morphology and phenotype. However, PMN-MDSCs express M-CSFR and a CD244 molecule with higher ARG1 activity and lower phagocytic activity than neutrophils [32]. They can be separated by density gradients, with PMN-MDSCs in the low-density Ficoll gradient fraction of peripheral blood mononuclear cells (PBMCs) and neutrophils in the high-density fraction [33]. Additionally, one study indicated that LOX-1 could distinguish the population of human PMN-MDSCs from granulocytes, which is thought to be a marker of human PMN-MDSCs [34]. Since monocytes are CD14^+^CD15^−^ HLA-DR^+^ and M-MDSCs are CD14^+^ HLA-DR^−^, human M-MDSCs can be isolated based on the presence of MHC class II molecules [33]. Table 2.

**Table 1:** The phenotypes of murine PMN-MDSCs, neutrophils, M-MDSCs and monocytes

	PMN-MDSCs	Neutrophils	M-MDSCs	Monocytes
CD11b	+	+	+	+
Ly6G	+	+	–	–
Ly6C	–	–	+	+
Gr1	+/high	+	+/low	+
F4/80	–	–	+/−	+
CD84	+	+	–	–
CD49d	–	–	+	–

**Table 2:** The phenotypes of human PMN-MDSCs, neutrophils, M-MDSCs and monocytes

	PMN-MDSCs	Neutrophils	M-MDSCs	Monocytes
CD11b	+	+	+	+
CD14	–	–	+	+
CD15	+	+	–	–
CD66b	+	+	–	–
HLA-DR	–	+	−/low	+
CD33	+	+	+	+
IL-4Rα (CD124)	–	–	+	+
CD16	–	+	–	–
CD84	+	+	–	–
LOX1	+	–	–	–

Although research on MDSCs has spanned for decades, key questions remain as to whether these cells are the precursors of well-established normal myeloid cells and whether there are other unidentified myeloid subpopulations of these cells [35].

## Expansion and activation of MDSCs

The conflicting results described above have been reported in a few other studies. The amplification process of MDSCs is complex, and the exact process of how MDSCs are generated from bone marrow and eventually become a population of cells with immunosuppressive function has become a highlight of this field.

**Fig. 2:**
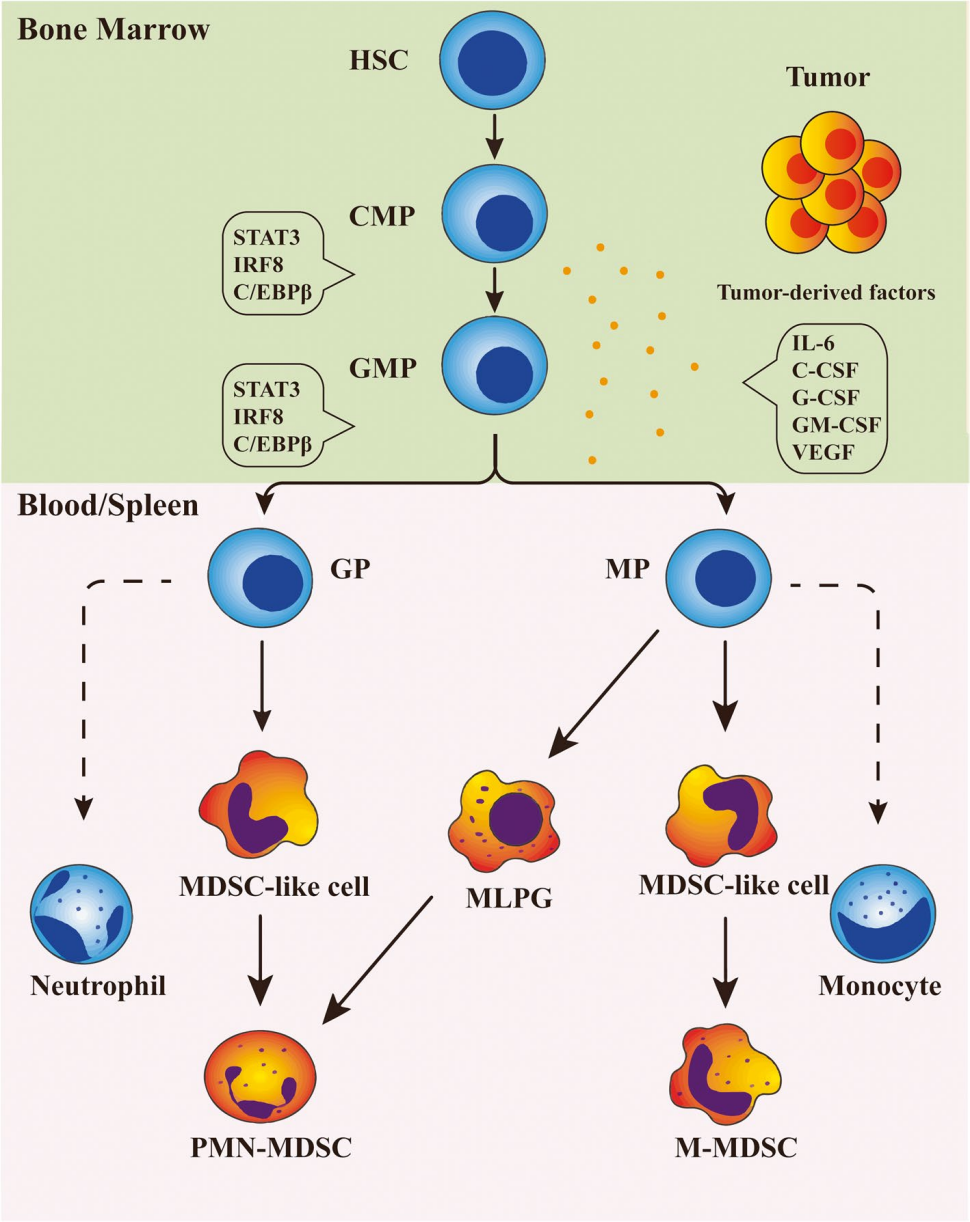
The origin of MDSCs. MDSCs are derived from HSCs, common myeloid progenitors (CMPs) and granulocyte–macrophage progenitors (GMPs). GMPs then differentiate into granulocyte progenitors (GPs) and monocytic progenitors (MPs). In response to multiple tumor-induced cytokines, MDSCs were developed through signaling pathways such as STAT3, IRF8 and C/EBPβ

MDSCs are derived from HSCs, common myeloid progenitors (CMPs) and granulocyte-macrophage progenitors (GMPs ) [36]. GMPs then differentiate into granulocyte progenitors (GPs) and monocytic progenitors (MPs) in response to multiple tumor-induced growth factors, cytokines and other factors [37] **(**Fig. 2). Although the molecular mechanisms of MDSCs expansion have been intensively studied over the years, the exact details remain unclear. An increasingly large number of scholars favor the two-signal model, which suggests that the generation of MDSCs is a sequential but overlapping process induced by two different signal transduction pathways [38]. One pathway dominates the proliferation of MDSCs, whereas the second pathway contributes to MDSCs activation.

### MDSCs expansion

**Fig. 3:**
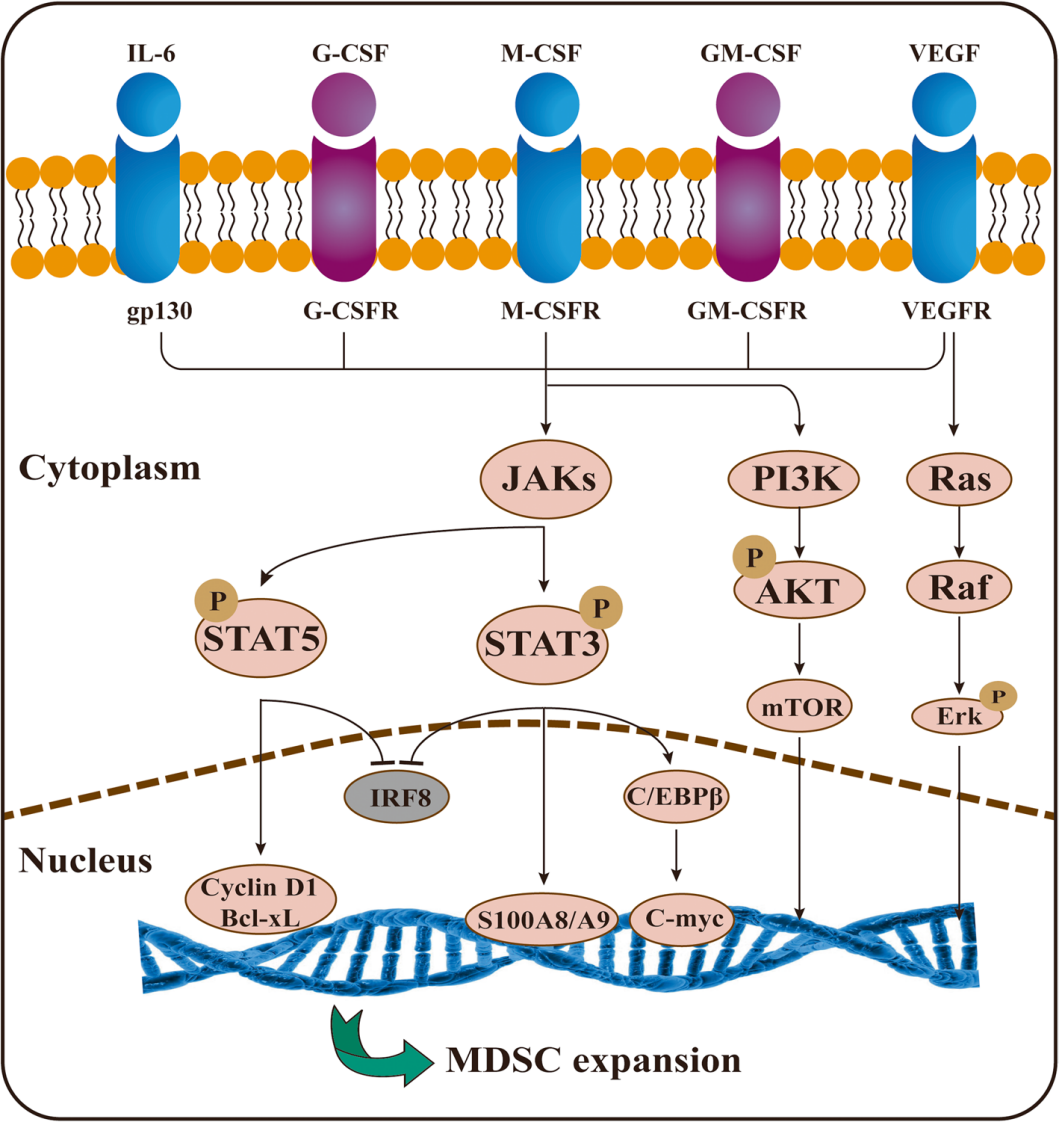
The mechanism of MDSCs expansion. The proliferation of MDSCs is mostly driven by GM-CSF, G-CSF, M-CSF, VEGF and IL-6. Additionally, several downstream factors are involved in regulating MDSCs expansion. Of particular interest is S100A9, STAT3, C/EBPβ and IRF8

Previous studies have shown that the proliferation of MDSCs is mostly driven by tumor-derived growth factors, which include GM-CSF, G-CSF, M-CSF, VEGF and IL-6 [39] **(**Fig. 3). Under physiological conditions, GM-CSF promotes myelopoiesis, and G-CSF and M-CSF are both in charge of differentiation [40]. While it was known as early as 1999 that GM-CSF alone is capable of eliciting these inhibitory cells [41], the details of how GM-CSF triggers an increase in MDSCs remained poorly understood until a preclinical experiment indicated that GM-CSF amplified GMP and was the main factor promoting the CD11b^+^MDSCs immunosuppressive pathway [42]. Further studies revealed that GM-CSF predominantly invoked Gr-1 ^int/low^ MDSCs, while G-CSF drove the proliferation of Gr-1^high^ MDSCs [42]. Another study demonstrated that G-CSF favored the production of MDSCs and whole-body amplification in a mouse breast cancer model [36]. VEGF severely impairs DC maturation and is responsible for the amplification of MDSCs [43]. IL-6 has been found to be positively correlated with peripheral blood MDSCs levels. Notably, all these experiments were conducted in vitro. Almand et al. measured the plasma concentrations of six cytokines, M-CSF, GM-CSF, IL-6, IL-10, TGF-β, and VEGF, in patients with head and neck squamous cell carcinoma (HNSCC), NSCLC, and breast cancer. They discovered that only elevated VEGF levels were statistically correlated with the expansion of MDSCs [44].

The cytokines discussed above then trigger several transcription factors, mainly signal transducer and activator of transcription 3 (STAT3), C/EBPβ and IRF8 [26, 38].

### STAT3

STAT3 was the first transcription factor found to be associated with MDSCs amplification in tumors. STAT3 upregulates the expression of the antiapoptotic genes Bcl-xL, c-myc and cyclin D to disrupt the normal differentiation of myeloid cells, thus augmenting the population of MDSCs [45, 46]. Additionally, STAT3 can directly bind to the ARG1 promoter to increase ARG1 expression and ROS production [47]. Previous studies have confirmed that the hyperactivation of the JAK–STAT3 pathway mediated the abnormal differentiation of DCs and thus increased the accumulation of MDSCs [48, 49]. In addition to upregulation, STAT3 downregulation caused by activated CD45 phosphatase was found in M-MDSCs, leading to a unique result of differentiation into TAMs [50]. In an inducible STAT3 knockout mouse model, multiple immune cell lineages showed enhanced antitumor activity when tested individually [51]. JSI-124, a selective JAK/STAT3 inhibitor, significantly reduced the presence of MDSCs and promoted their differentiation, suggesting that the inhibition of JAK/STAT3 signaling overcame the differentiation block [52]. However, the studies mentioned above utilized MDSCs in vitro or from cells isolated from lymphoid organs. Kumar et al. found that STAT3 activity of MDSCs is relatively lower in tumor sites than in the spleen and blood of patients [50]. Moreover, inhibition of STAT3 decreased splenic MDSCs, but no significant change in MDSCs was found in the tumor site [53]. Using a spontaneous medulloblastoma transgenic murine model, the prevalence of PMN-MDSCs was reduced after STAT3 disruption, but the number of M-MDSCs increased instead of decreased [54]. Therefore, the mechanistic details of how STAT3 impacts MDSCs in the TME require more intensive investigation.

In addition, several factors downstream of STAT3 may be engaged in regulating MDSCs expansion. Of particular interest is the proinflammatory protein S100A9 together with its dimerization partner S100A8, which is strongly upregulated in multiple tumors, including colon, breast, and prostate cancers [55]. S100A8/A9 directly binds to p47^phox^ and p67^phox^, enhancing the activation of NOX2 and thus leading to increased ROS production to increase inhibitory functions [29]. S100A8/A9 was also shown to bind to a receptor located on the MDSCs membrane to promote MDSCs migration. After blocking the conjugation of S100A8/A9 to its receptor, the number of MDSCs in peripheral blood was found to be decreased [56]. Another experiment found no amplification of MDSCs in the peripheral blood or spleen of S100A9-deficient tumor-bearing mice, confirming previous findings [57]. In contrast, overexpression of S100A9 in mice resulted in the accumulation of MDSCs, and increased secretion of IL-10 and TGF-β [58]. Taken together, these experiments illustrate the vital role of S100A8/A9 in MDSCs amplification. However, the specific details remain to be explored more deeply.

### C/EBPβ

CCAAT/enhancer-binding protein (C/EBP) β, a member of the C/EBP transcription factor family, is implicated in cell proliferation, differentiation and apoptosis [59, 60]. C/EBPβ has fundamental roles in myelopoiesis and emergency granulopoiesis, the level of which increases excessively at later stages of myeloid differentiation [61, 62]. IL-6-mediated C/EBPβ downregulates the expression of immunosuppressive genes such as ARG1, iNOS and NOX2, thus regulating MDSCs differentiation and function [63]. C/EBPβ has been reported to contribute to the generation of MDSCs in the bone marrow and spleen by activating microRNA-21 and microRNA-181b expression [64]. C/EBPβ is also closely associated with GM-CSF and G-CSF expression in myeloid cells and regulates the immunoregulatory activity of MDSCs [65, 66]. Moreover, Strauss et al. found that retinoic acid-related orphan receptor 1 (RORC1) orchestrated myelopoiesis by promoting C/EBPβ and PMN-MDSCs accumulation [67]. In contrast, C/EBPβ-deficient mice have decreased splenic CD11b^hi^MDSCs. Surprisingly, M-MDSCs were the most reduced population, indicating that the main vital impact of C/EBPβ is on the differentiation of M-MDSCs [68]. There is a consensus that C/EBPβ is indispensable for MDSCs proliferation. However, the exact stage at which C/EBPβ particularly affects MDSCs still needs to be addressed.

### IRF8

Interferon regulatory factor-8 (IRF-8), also called interferon consensus sequence binding protein (ICSBP), is crucial for normal myelopoiesis. Mice with a null mutation of IRF-8 exhibit deregulated hematopoiesis, ultimately leading to chronic myelogenous leukemia [69]. Unexpectedly, a previous experiment demonstrated that IRF-8-deficient mice exhibited remarkable accumulation of MDSCs. In another study using both implantable and transgenic mouse models, IRF-8 was observed to play an integral role in the tumor-induced expansion of MDSCs [70]. In addition, IRF8 also functions as a negative regulator in human MDSCs of breast cancer patients. Downregulation of IRF-8 was demonstrated to induce PMN-MDSCs production. In vivo IRF-8 overexpression specifically attenuated MDSCs expansion and enhanced antitumor efficacy via the STAT3 and STAT5 signaling pathways [71, 72]. Notably, IRF-8 promotes monocyte and dendritic cell differentiation but limits granulocyte development [73]. A recent study demonstrated that IRF8 overexpression in vivo selectively led to GPs proliferation and PMN-MDSCs expansion without appreciable expansion of MPs and M-MDSCs [71].

### MDSCs activation

**Fig. 4:**
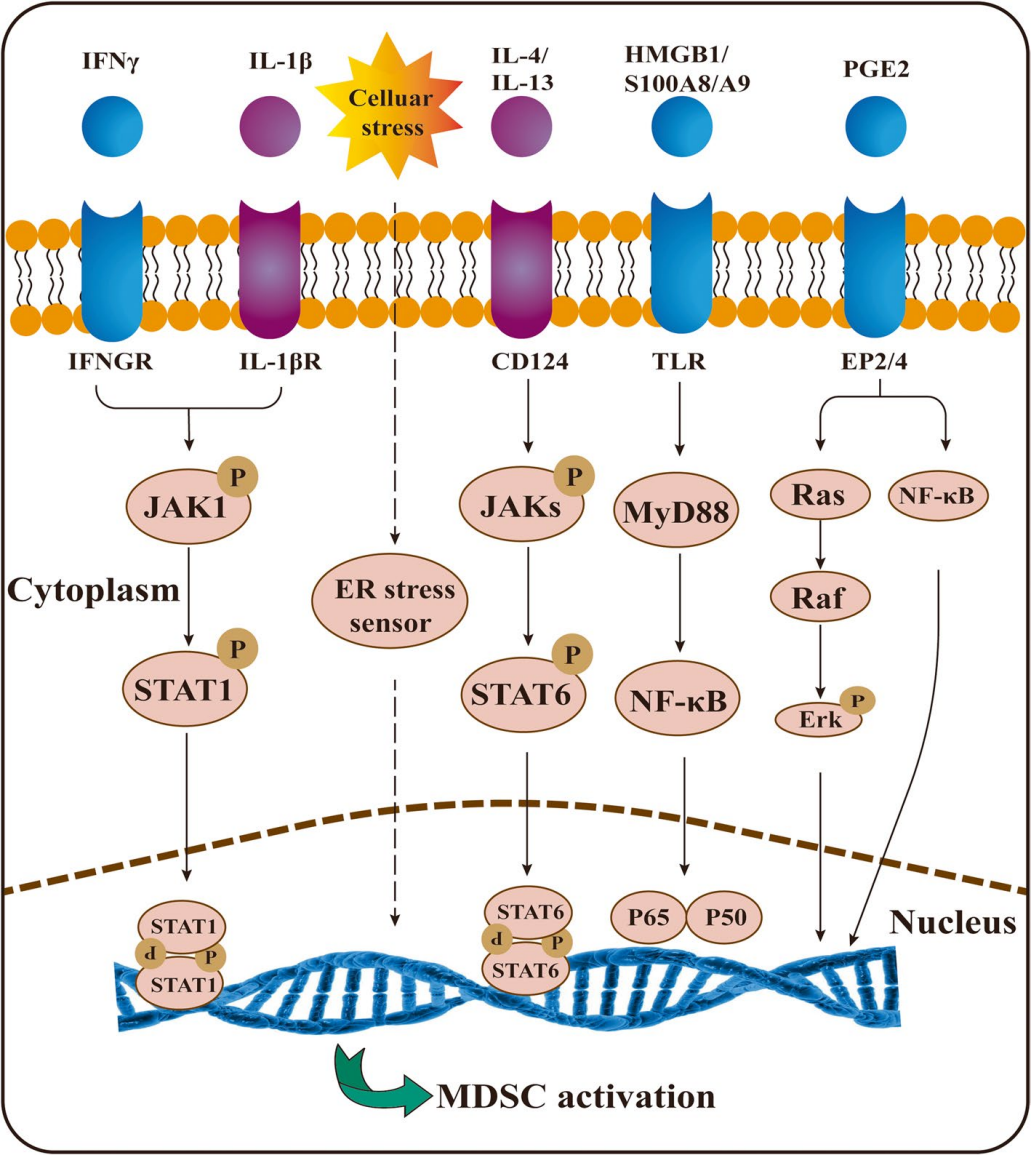
The mechanism of MDSCs activation. Notably, only after activation can MDSCs acquire immunosuppressive activity. MDSCs activation is primarily associated with IFN-γ, IL-1β, IL-4, IL-13, PGE2 and ER stress sensors. The signaling pathways involved in MDSCs activation include STAT6, nuclear factor-κB (NF-κB) and STAT1

Notably, MDSCs acquire immunosuppressive activity only after activation. The second signal governing MDSCs activation is primarily proinflammatory cytokines produced by the tumor stroma or activated T cells, including IFN-γ, IL-1β, IL-4, IL-13, and PGE2. The signaling pathways involved in MDSCs activation include STAT6, nuclear factor-κB (NF-κB) and STAT1 [29] (Fig. 4).

IFN-γ is released by CD3/28-triggered activation of T cells, and due to the presence of IFN-γ, MDSCs become immune dysfunctional [74]. STAT1 is the most crucial downstream transcription factor of IFN-γ. Indeed, IFN-γ is strictly required for the activation and function of PMN-MDSCs, and is dependent on the STAT1 pathway or NO production. However, blocking IFN-γ only partially antagonizes the immune dysfunction of M-MDSCs [30]. IFN-γ and IL-13 were found to synergistically initiate immunosuppressive pathways of MDSCs. IFN-γ preferentially promotes iNOS expression, while IL-13 has a greater tendency to upregulate ARG1 [25]. Importantly, both enzymes are upregulated when IL-13 and IFN-γ are simultaneously or sequentially added. The inflammatory mediator IL-1β was shown to be a cytokine that induced the recruitment of MDSCs and, in particular, promoted the activation of MDSCs. Another study demonstrated that the PGE2 receptor expressed in MDSCs induced ARG1 expression, and using a COX2 inhibitor decreased the level of ARG1 in vitro and in vivo [75]. PGE2 is indispensable for the functionality of MDSCs. Indeed, blocking COX-2, an enzyme that catalytically synthesizes PGE2, potently revived the ability to suppress T-cell function mediated by MDSCs [76, 77]. Tumor-derived PGE2 has been shown to drive the suppressive phenotype of M-MDSCs through upregulation of NF-κB [78]. Moreover, PGE2 activated the Ras/Erk pathway and increased the level of TGF-β to activate the suppressive functions of MDSCs on NK cells [79].

Upregulation of genes associated with the ER stress response is a prominent feature of MDSCs. The ER stress response is highly conserved and serves to defend cells from a variety of emergency damages, such as hypoxia and infection [80]. MDSCs isolated from tumor-bearing mice and cancer patients were identified to upregulate downstream effectors of the ER stress response, especially C/EBP-homologous protein (CHOP ) [81]. Another study showed that CHOP deficiency impaired inhibitory activity in MDSCs and decreased the expression of IL-6, C/EBPβ, and pSTAT3. Additionally, exogenous IL-6 rescued MDSCs activity in Chop-deficient mice [82]. Consistent with these observations, the administration of ER stress inducers increased the expansion of MDSCs and their inhibitory function.

Recently, HMGB1 and PPARγ were found to exert an important role in the function of MDSCs [39]. HMGB1 released by MDSCs was reported to, by activating NF-κB, promote the differentiation of MDSCs, increase secretion of IL-10, and decrease the expression of L-selectin on circulating T cells, exerting immunosuppressive effects [83]. Overexpression of PPARγ led to the expansion of PMN-MDSCs with immunosuppressive activity, which was also associated with the NF-κB pathway [84].

## Immunosuppressive mechanism of MDSCs

**Fig. 5:**
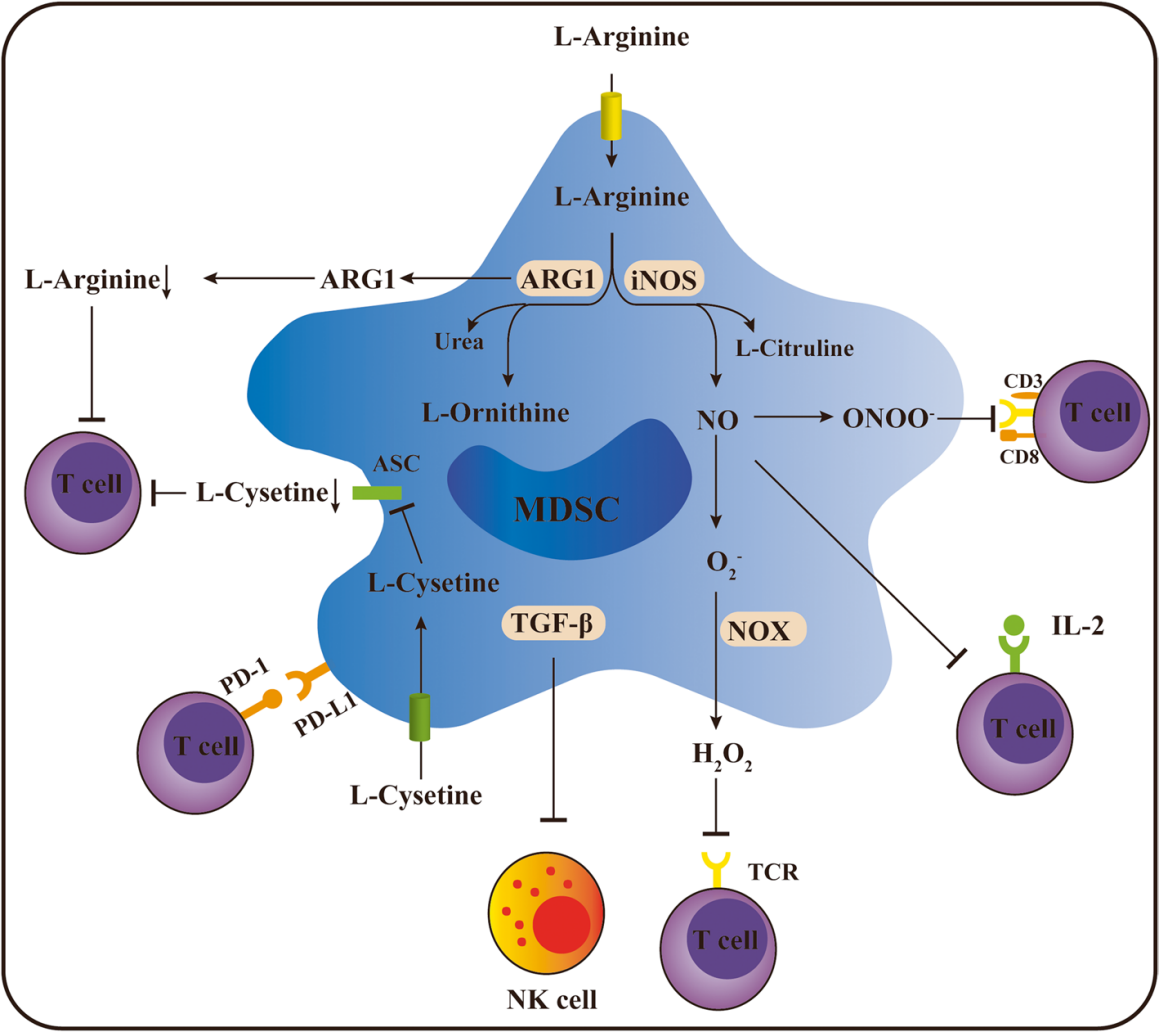
The mechanisms by which MDSCs inhibit T-cell antitumor immunity. MDSCs exert their T-cell suppression mainly via the high expression of ARG1 and iNOS and the production of ROS. In addition to biochemical metabolism, MDSCs also induce immunosuppression by upregulating PD-L1 expression and secretion of TGF-β

Previous studies have demonstrated that MDSCs specifically and effectively inhibit antigen-specific CD8^+^ T-cell function with decreased IFN-γ production, and this effect is dependent on the interaction between MDSCs and the T cells [85, 86]. Using immortalized murine CD11b ^+^ /Gr1 ^+^ cells, Bronte et al. found that MDSCs reduce the generation of T cells and suppress tumor immunity by triggering the apoptotic cascade of T cells [87]. They followed up their study by demonstrating that MDSCs halted the cell cycle of T cells, leading to apoptosis through proliferation blockade, rather than directly killing the cells [88]. Summarizing experimental murine models and clinical findings to date, MDSCs exert their T-cell suppression mainly through the high expression of ARG1, iNOS and ROS [89–94] (Fig. 5).

### ARG1

Two arginine isozymes exist in mammals. ARG1, abundantly observed in the cytoplasm of hepatocytes, is an important element in the urea cycle. In comparison, ARG2 exists in mitochondria and is barely expressed in the liver [95]. MDSCs express high levels of ARG1, rather than ARG2, which is induced by Th2-type cytokines such as IL-4 and IL-13 [96]. ARG1 catalyzes the synthesis of urea and L-ornithine from L-arginine, the latter being an essential substrate for cell cycle processes. Therefore, extracellular L-arginine, an essential amino acid for T-cell activation, is substantially diminished [97]. Except in the case of T-cell anergy, L-arginine depletion decreases the expression of the CD3-associated ζ chain, suppressing T-cell proliferation [98–100]. Interestingly, one study demonstrated that MDSCs in the spleen downregulated the CD3-associated ζ chain of CD4^+^ T cells but not CD8^+^ T cells [101]. To investigate the mechanisms by which L-arginine deletion induces the inability of T cells to proliferate，Rodriguez et al. found that L-arginine starvation arrested the cell cycle from G1 to S phase by impairing the expression of cyclin D [102]. These data are consistent with the observation that MDSCs producing high ARG1 exist in patients with renal cell carcinoma and express lower levels of T-cell receptor (TCR) and CD3-associated ζ chain [103], indicating that ARG1 plays a critical role in immunosuppression of MDSCs both in mice and in patients. In addition, depletion of cystine and cysteine is also involved in the immunosuppressive effect of MDSCs. MDSCs importing cystine but not releasing cysteine restrict the levels of cysteine in the TME, thus limiting T-cell activation [104].

### iNOS

There are three isoforms of nitric oxide synthase (NOS), neuronal NOS (nNOS), endothelial NOS (eNOS), and iNOS [105]. The first two isoforms are constitutively expressed. In contrast, iNOS is only expressed when stimulated and is highly associated with poor prognosis in malignant cancers [106]. iNOS competes for the same substrate as ARG1 and metabolizes L-arginine to citrulline and NO, which is a key messenger in tumor progression and T-cell activation [107, 108]. NO generated by MDSCs was found to abolish the IL-2 receptor signaling pathway and nitrate the TCR, resulting in immunosuppressive activity [109]. However, unlike ARG1 induced by Th2 cytokines, iNOS is induced by Th1 cytokines such as IFN-γ, TNF-α and IL-1 β [110]. This confirms the previous finding that blockade of IFN-γ eliminates the complete suppression of PMN-MDSCs and partial effect of M-MDSCs [30]. The intracellular signal transduction pathways involved are the NF-κB and JAK-STAT pathways [111]. Moreover, a new possible mechanism was proposed in which iNOS critically downregulated vascular E-selectin, impairing T-cell recruitment to tumors and antitumor immunity [112]. Similarly, the chemokine receptor CCR2 expressed by MDSCs and CCL2 produced by tumor cells have been implicated in a vital role in the recruitment of MDSCs into tumors [113]. iNOS^+^ MDSCs cause CCL2 nitration and inhibit T-cell migration.

Recent studies have revealed that the network of the two enzymes, ARG1 and iNOS, working together may be unique to MDSCs [114]. ARG1 overexpression led to a translational arrest of the mRNA for iNOS and reduced iNOS activity; while iNOS is overexpressed, ARG1 was in turn inhibited, and NO was further released in adjacent cells [114].

### Reactive oxygen species (ROS)

Previous studies have provided multiple lines of evidence supporting the critical role of ROS in MDSCs immunosuppression [115, 116]. The ROS levels of MDSCs isolated from tumor-bearing mice were found to be significantly higher than those isolated from healthy mice. The main producer of increased ROS is NADPH oxidase (NOX2), which is composed of two membrane proteins and at least four cytosolic proteins, including p47^phox^ and p67^phox^ [117]. Several dramatically increased NOX2 subunits, directly regulated by STAT3, were found to result in ROS production [118]. The biochemical metabolisms of MDSCs produce ROS, including superoxide (O2^−^), hydrogen peroxide (H_2_O_2_), and peroxynitrite (ONOO^−^) [6]. H_2_O_2_ is a major contributor to this increased pool of ROS. Indeed, inhibiting ROS in MDSCs completely reversed MDSCs immunosuppression, suggesting that MDSCs suppress the CD8^+^ T-cell response via the production of ROS [119]. Moreover, H_2_O_2_ produced by MDSCs also had an impact on CD3-associated ζ chain expression and function [120]. ONOO^−^ also causes DNA damage as well as nitration of various proteins, such as TCR, CD3 and CD8 [117].

In addition to the biochemical metabolisms discussed above, MDSCs also induce immunosuppression by upregulating PD-L1 expression and secretion of TGF-β. The percentage of PD-L1 expression is noticeably higher on tumor-infiltrating MDSCs than splenic MDSCs [121]. Another study demonstrated that tumor-derived PD-L1 expression was limited to M-MDSCs and that these cells directly eliminated CD8^+^ T cells in vitro [122]. Moreover, in the presence of MDSCs, the surface molecules of B cells are remodeled, with prominently increased PD-L1 expression, subsequently inducing T-cell dysfunction [123]. MDSCs were found to potently inhibit NK-cell cytotoxicity, which requires direct intercellular contact to suppress perforin production rather than granzyme B [124]. In orthotopic live tumor models, hepatic NK-cell cytotoxicity and secreted IFN-γ were remarkedly damaged. Furthermore, MDSCs induce NK-cell anergy via membrane-bound TGF-β [125]. However, Nausch et al. unexpectedly found that F4/80^+^MDSCs in mice could instead initiate NK cells and augment IFN-γ secretion [126].

## The plasticity of MDSCs

**Fig. 6:**
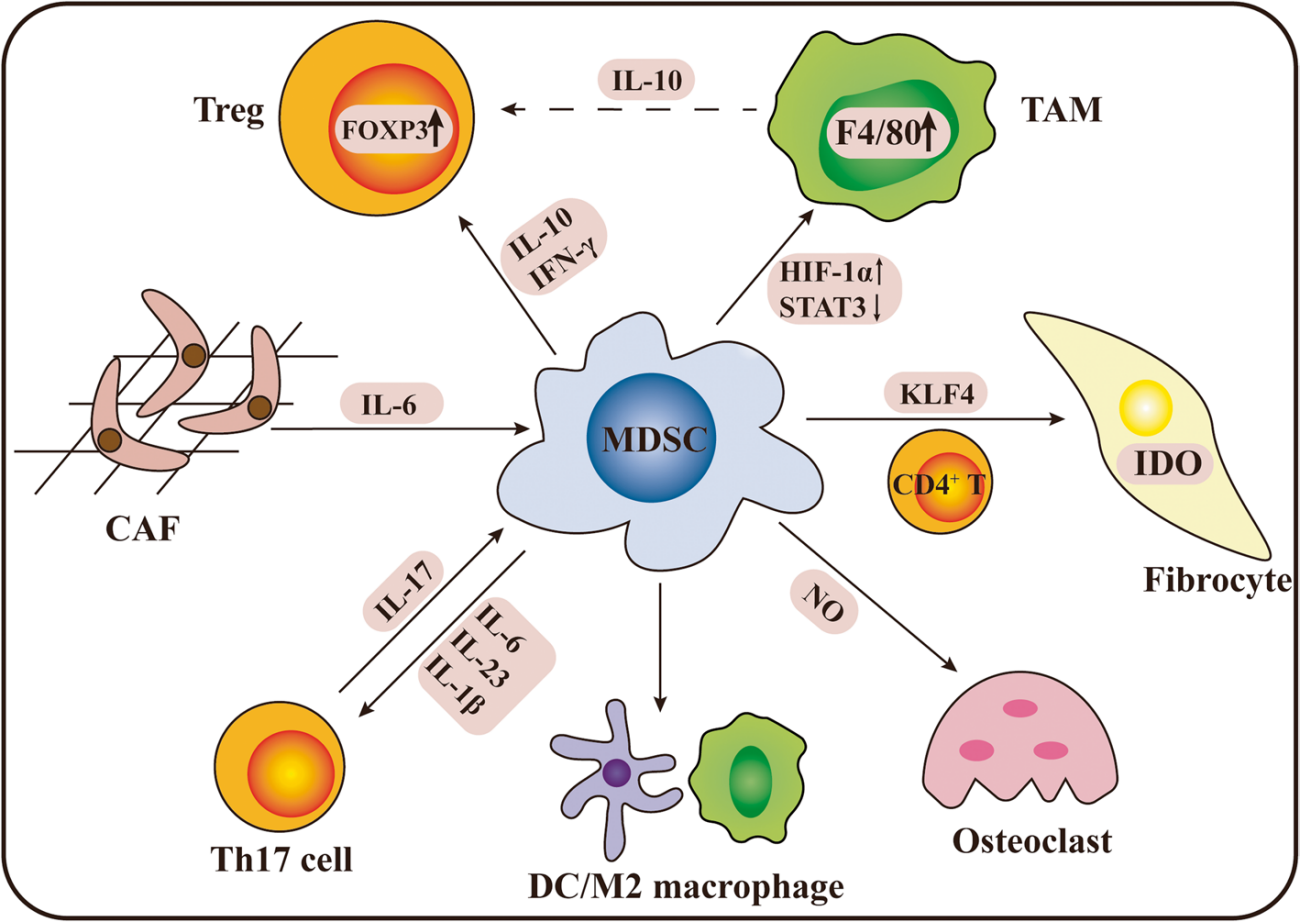
The plasticity of MDSCs. MDSCs can induce Tregs and differentiate into TAMs, as well as mature DCs and M2 macrophages. Moreover, MDSCs were found to be derived from CAFs under the influence of IL-6 and turned into fibrocytes in the lung. MDSCs can also differentiate into osteoclasts and have a complex crosstalk with Th17 cells

Plasticity is a distinct characteristic of MDSCs (Fig. 6). In the last few years, three major cell populations have gained attention as major negative regulators of the immune response: tumor-associated macrophages (TAMs), MDSCs, and CD4^+^ regulatory T cells (Tregs) with the same immunoinhibitory functions that limit the effectiveness of ICI therapy [127–132]. MDSCs can influence the proliferation of Tregs, inhibiting T-cell activation. Tregs represent a group of special T cells that are divided into two main groups: natural Tregs and adapted Tregs. The former is produced in the thymus and is developmentally dependent on the expression of the forkhead box transcription factor (FOXP3). CD115^+^ F4/80^+^ MDSCs, cocultured with IFN-γ and IL-10, were shown to induce FOXP3^+^ Tregs in vivo [11].

TAMs are the second most well-described myeloid cell population negatively affecting immunotherapy. The relationship between TAMs and MDSCs has not been fully established, but TAMs may be partially derived from MDSCs [110]. Compared to TAMs, MDSCs exhibit high Gr1 expression and low F4/80 expression. After migration to a tumor site, MDSCs are capable of differentiating into TAMs, limiting the efficacy of the immune response by inducing T-cell apoptosis [133, 134]. MDSCs isolated from the spleen of tumor-bearing mice arrive at the tumor site and became F4/80^+^ TAMs, characterized by constitutive expression of ARG1 and iNOS [133]. A recent study found that HIF-1α was a key component of this differential process [135]. Hypoxia selectively increase the expression of PD-L1, an extremely important target in ICIs, in MDSCs via HIF-1α directly binding to the PD-L1 proximal promoter [121]. Moreover, recent reports suggested that significant downregulation of STAT3 is a major factor in regulating this kind of differentiation. Hypoxia-induced CD45 protein tyrosine phosphatases caused upregulation of HIF-1α and downregulation of STAT3, facilitating the differentiation of M-MDSCs into TAMs [50]. Another study indicated that circulating M-MDSCs were essential for TAMs accumulation [136]. Additionally, M-MDSCs can differentiate into mature macrophages and DC [137]. Ginderachter et al. demonstrated that M-MDSCs in the spleen could turn into M2 macrophages [138]. Inhibition of STAT3 in MDSCs facilitates their conversion to mature DCs [139].

It is well known that IL-17 is the key cytokine produced by Th17 cells [140]. One study found that MDSCs producing IL-1β, IL-6, and IL-23 promoted the differentiation of Th17 cells, which is critically NO-dependent [141]. Furthermore, innate γδT17 cells were demonstrated to be the major cellular source of IL-17, promoting the accumulation of MDSCs in human colorectal cancer [142].

Cancer-associated fibroblasts (CAFs) can polarize monocytes and convert them into MDSCs by increasing oxidative stress, with suppression of CD8^+^ T-cell activity and the production of IFN-γ. Furthermore, in the presence of a NOX2 inhibitor, CAF-induced MDSCs were found to attenuate ROS production and restore antitumor immunity [143]. Additionally, MDSCs have the ability to nonspontaneously differentiate into fibroblasts in the lung under the influence of CD4^+^ T cells [144]. Knockout of KLF4, a transcription factor that is crucial to monocyte differentiation and tumor development, decrease the generation of MDSCs and MDSC-derived fibrocytes in the lung, reducing pulmonary metastasis [145]. Indeed, these fibrocytes, mediating immune suppression via indoleamine oxidase (IDO) and Tregs expansion, have been described as a novel subset of cancer-induced MDSCs in patients with metastatic cancer [146, 147]. In addition, a study using a bone metastasis mouse model suggested that MDSCs are capable of differentiating into functional osteoclasts both in vivo and in vitro, mechanistically dependent on NO [148].

## Harnessing MDSCs for therapy

**Fig. 7:**
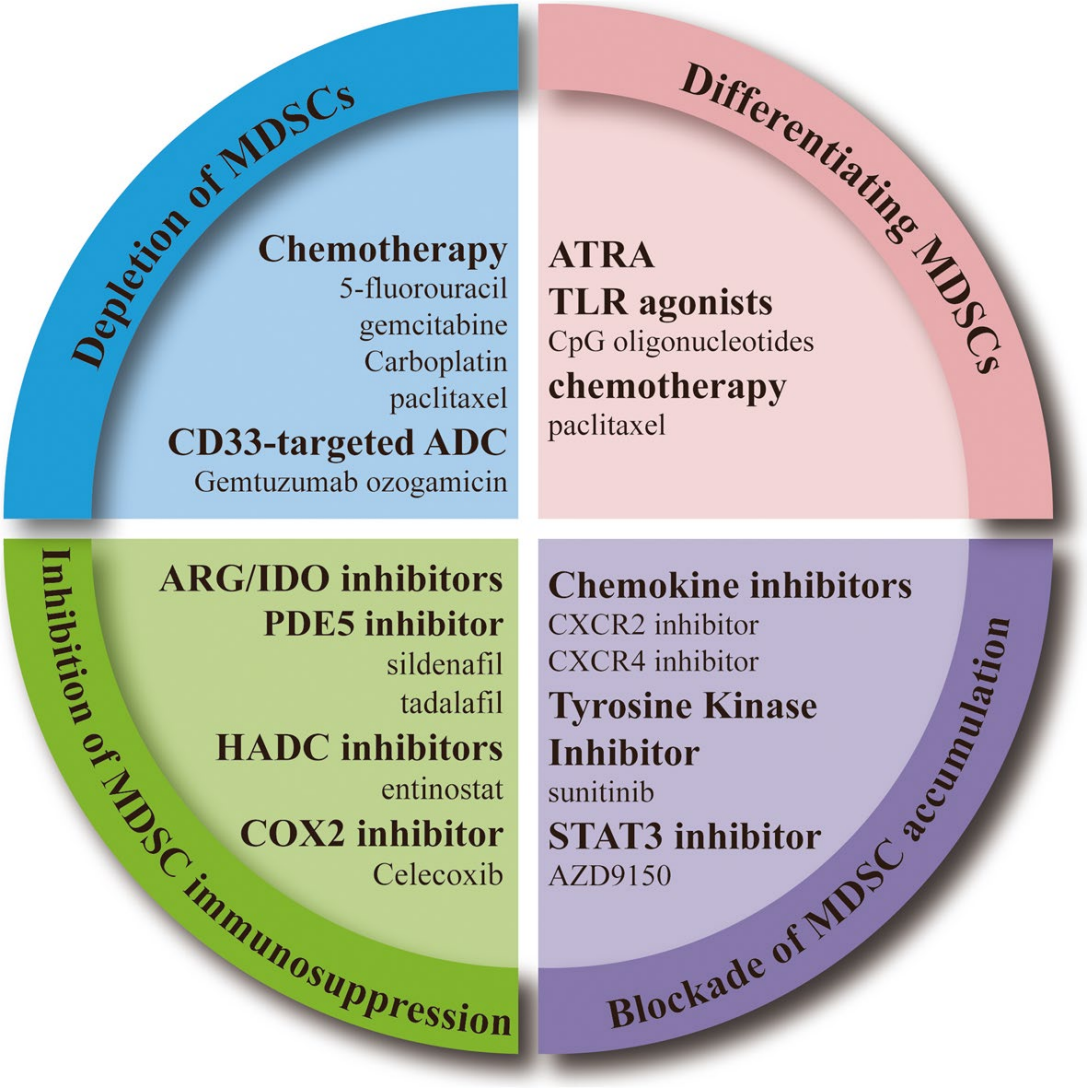
The MDSC-targeting therapeutic strategies. Current clinical therapies targeting MDSCs are mainly focused on four aspects: depleting MDSCs, differentiating MDSCs, inhibiting MDSCs immunosuppressive activity, and blocking MDSCs expansion or activation

MDSCs perform an essential function in tumor-associated immune suppression, which subsequently greatly limits the therapeutic effectiveness of cancer immunotherapy [149]. Therefore, how to eliminate these cells and reconstitute the immunosuppressive microenvironment has become a focus of research in this field. Current clinical therapies targeting MDSCs are mainly focused on four aspects: depleting MDSCs, differentiating MDSCs, inhibiting MDSCs immunosuppressive activity, and blocking MDSCs expansion or activation [150, 151] (Fig. 7).

### Depletion of MDSCs

Chemotherapy can eliminate immunosuppressive cells from the TME [152]. Gemcitabine and 5-fluorouracil selectively induced the apoptosis of MDSCs in the spleen and tumor site and enhanced the antigen-specific production of IFN-γ by intratumor CD8^+^ T cells [153]. Carboplatin and paclitaxel reduced the abnormally increased circulating MDSCs and fostered vigorous antitumor responses in advanced cervical cancer patients [154]. However, these agents are not specific to MDSCs and affect all rapidly proliferating cells, including effector T cells.

Gemtuzumab ozogamicin (GO) is a CD33-targeted antibody–drug conjugate (ADC) linked to calicheamicin, that specifically targets the membrane antigen CD33 and releases a derivative of the cytotoxic calicheamicin component after internalization, leading to tumor cell death [155, 156]. GO has been approved for the treatment of CD33^+^ acute myeloid leukemia and had an acceptable safety profile in multiple clinical trials [157–159]. Although human PMN-MDSCs and M-MDSCs are transcriptomically distinct, CD33 is a common target for MDSCs regardless of subtype. GO was found to increase the death of MDSCs, providing a clinically plausible approach to deplete MDSCs in cancer patients [160].

### Differentiating MDSCs

MDSCs differentiation is regulated by complex signals, but the specific regulatory mechanisms are not well understood. All-trans retinoic acid (ATRA) was previously found to show potent activity on MDSCs [85]. In vivo administration of ATRA significantly decreased the presence of MDSCs by differentiating MDSCs into mature myeloid cells [161]. ATRA was also shown to abrogate MDSC-mediated immunosuppression [162, 163]. To determine the mechanism of this effect, Nefedova et al. found that ATRA specifically upregulated the gene expression of glutathione synthase (GSS) and glutathione (GSH) accumulation in MDSCs [116]. Indeed, inhibiting GSH synthesis blocked the effect of ATRA on MDSCs [116]. In preclinical models, ATRA was shown to remove MDSCs for improvement of efficacy of antiangiogenic therapies in breast cancer [164]. Ipilimumab, a fully humanized antibody targeting cytotoxic T-lymphocyte antigen-4 (CTLA-4), was the first approved therapy in advanced melanoma patients [165]. However, melanoma wields substantial immunosuppressive mechanisms, especially an increase in circulating MDSCs, limiting the efficacy of ipilimumab [166]. In a randomized phase II clinical trial, ipilimumab monotherapy or ipilimumab plus ATRA was used to treat patients with advanced melanoma. ATRA combined therapy was found to significantly reduce the frequency of circulating MDSCs with a safe profile (NCT02403778 ) [167]. However, the poor solubility and fast metabolism of ATRA limits its applications in cancer immunotherapy. HF1K16, a pegylated liposome formulation of ATRA with a great dose loading capacity and sustained drug release property, is under phase I clinical trial for its safety and tolerability (NCT05388487).

In addition, CpG oligonucleotides (ODNs), a Toll-like receptor 9 (TLR9) agonist, directly induces the activation of the immune response [168]. One study examined the effect of CpG ODN on MDSCs and found a decline in the frequency and inhibitory activity of M-MDSCs partly for inducing the differentiation of M-MDSCs into M1-like macrophages [169]. IFN-α stimulated by CpG is a key effector for the induction of MDSC maturation in vitro [170]. Notably, the codelivery of CpG ODN and TLR7/8 agonists more significantly reduced the frequency of M-MDSCs compared with monotherapy [171]. Another study showed that low concentrations of paclitaxel neither increased MDSCs apoptosis nor blocked MDSCs generation but stimulated MDSCs differentiation toward mature DCs [172].

### Inhibition of MDSCs immunosuppression

Targeting the biochemical pathways of MDSCs, such as with ARG1, iNOS, COX2 and TGF-β inhibitors, is a good strategy to improve the effectiveness of various immunotherapies [173]. In several mouse tumor models, the phosphodiesterase-5 (PDE5) inhibitor sildenafil, tadalafil and vardenafil downregulates the expression of ARG1 and iNOS, reversing MDSC-induced immunosuppression and restoring antitumor immunity [173, 174]. Subsequently, sildenafil has also been shown to reduce MDSCs in a transgenic mouse melanoma model [175]. Moreover, multiple clinical trials have demonstrated that tadalafil reduces MDSCs concentrations and augments general and tumor-specific immunity in both HNSCC and metastatic melanoma patients [176–178].

Recently, the impact of histone deacetylase inhibitors (HADCi) on MDSCs has attracted a great deal of attention [179, 180]. A corrective analysis of a randomized, phase II trial in patients with breast cancer demonstrated that entinostat, a class I HADCi, decreased the frequency of circulating PMN-MDSCs and M-MDSCs [181]. A preclinical study showed that entinostat targeted MDSCs and increased the efficacy of ICIs in murine colorectal and breast cancers [182]. Entinostat reprogrammed tumor-infiltrating MDSCs by significantly inhibiting the expression of ARG1, iNOS and COX2 and suppressing the function of immunosuppressive MDSCs, thereby overcoming immune resistance [183, 184].

In addition, the COX2 inhibitor celecoxib blocks MDSCs suppressive function and delays tumor development by decreasing the expression of ARG1 [185]. Preclinical studies have shown that ARG inhibitors, which reverse the inhibition of T cells by blocking L-arginine depletion, reduce tumor growth in mouse models [186]. A phase I study (NCT02903914) was initiated to test the antitumor activity of ARG inhibitors alone or combined with anti-PD-1. IDO orchestrates immunosuppressive effects through recruitment and activation of MDSCs in a Treg-dependent manner. A selective IDO inhibitor was found in vivo to reverse tumor growth by decreasing the numbers of tumor-infiltrating MDSCs and abolishing their suppressive function [187].

### Blockade of MDSCs accumulation

Given the essential role of STAT3 in MDSCs accumulation, blocking STAT3 is a promising approach for MDSC-targeted immunotherapy. AZD9150, an antisense oligonucleotide designed to downregulate the expression of STAT3 mRNA, shows potent antitumor activity in patients with lymphoma and NSCLC [188, 189]. Preclinical data have provided evidence that AZD9150 accompanied with PD-L1 antibody displayed enhanced the antitumor activity [190]. These data provide a rationale for testing this combination in the clinic. AZD9150 is now being investigated in several phase I/II clinical trials in combination with ICIs.

In addition, clinical trials with sunitinib, a tyrosine kinase inhibitor, revealed that it could target MDSCs by blocking VEGF, a promotor for MDSCs expansion described above. Sunitinib significantly reduced MDSCs in patients with renal cell carcinoma [191]. Moreover, sunitinib was found to abrogate highly increased MDSCs, enhancing the efficacy of stereotactic body radiotherapy (SBRT) in patients with oligometastases [192].

MDSCs are recruited into the TME via interactions between chemokines and chemokine receptors [193]. M-MDSCs are recruited via CXCR4-CXCL12, CXCR2-CXCL5/CXCL8, and CCR2-CCL2 signaling, whereas CXCR1-CXCL8, CXCR2-CXCL8, and CCR5-CCL5 axes contribute to the recruitment of PMN-MDSCs [194, 195]. Targeting these chemokine receptors may prevent the accumulation of MDSCs in the TME. Inhibition of CXCR2 has been shown to rescue MDSCs trafficking and enhance anti-PD-1 efficacy in murine glioblastoma and rhabdomyosarcoma [196, 197]. Moreover, CXCR4 blockade has been shown to synergize with anti-PD-1 therapy in several mouse models [198]. A phase IIa, open-label, two-cohort clinical trial was conducted to assess the safety, efficacy and immunobiological effects of BL-8040, a CXCR4 antagonist. Notably, the CXCR4 antagonist decreased the number of MDSCs [199].

Benefiting from the advent of quantitative tools, such as single-cell RNA sequencing (scRNA-seq) and high-dimensional cytometry, additional phenotypes and therapeutic targets of MDSCs have progressed considerably [200]. In a mouse model of melanoma, GCN2, an environmental sensor controlling transcription and translation, was shown to be required for the phenotypes and function of MDSCs, making it an attractive target for decreasing MDSCs [201]. Depletion of GCN2 increased the inflammatory pathway with the strongest impact on the IL-1β pathway [201]. Another study found that MDSCs drove glioblastoma growth in a sex-specific manner. M-MDSCs could be targeted with antiproliferative agents in males, whereas IL-1β inhibitors were identified as potential drug candidates to target PMN-MDSCs in females. Strikingly, anti-IL-1β treatment counteracted PMN-MDSC-mediated immunosuppression and potently prolonged the survival of female mice, further providing the rationale for clinical testing of IL-1β inhibitors in cancer patients [31]. Using scRNA-seq, Alshetaiwi et al. delineated the molecular features of MDSCs in a mouse model of breast cancer and identified CD84 and JAML as several novel surface markers for faithful MDSC detection [202]. CD84 is a member of the signaling lymphocytic activation molecule (SLAM) family of cell-surface immunoreceptors, broadly expressed on most immune cell subsets [203]. JAML is a member of the junctional adhesion molecule (JAM) family and is expressed on neutrophils and monocytes [204].

Although these emerging quantitative tools provide numerous of previously unappreciated insights into the targets and biology of MDSCs, there are potential limitations here [205]. One limitation is that scRNA-seq may lack the surface protein information, thereby leading to misnamed or misclassified MDSC subtypes.

## Conclusion

The complexity of the tumor immune microenvironment has been gradually revealed by the combination of scRNA-seq and spatial omics [206, 207]. Not only the compositions and molecular features, but also the spatial architecture of immune components in the TME determine antitumor activity [208]. MDSCs, as a key component in the TME, are now recognized as an emerging target for anticancer immunotherapy, and their role in cancer development and treatment response is increasingly appreciated. However, due to ambiguous phenotypes, great heterogeneity and the complex network of origin and function, current therapeutic strategies targeting MDSCs are only partially effective [209]. There is a critical urgency to address the complexity and heterogeneity of MDSCs to develop novel clinical targets and strategies. In the coming years, it will be seen whether targeting MDSCs combined with ICIs may overcome the existing limitations of cancer immunotherapy.

## Data Availability

Not applicable.

## Abbreviations

MDSCs: Myeloid-derived suppressor cells
ICIs: Immune checkpoint inhibitors
TME: Tumor microenvironment
HSCs: Hematopoietic stem cells
DCs: Dendritic cells
IMCs: Immature myeloid cells
MSCs: Myeloid suppressor cells
PMN-MDSCs: Polymorphic nuclei-MDSCs
M-MDSCs: Mononuclear-MDSCs
MLPGs: Monocyte-like precursors of granulocytes
ARG1: Arginase I
iNOS: Inducible nitric oxide synthase
NO: Nitric oxide
ROS: Reactive oxygen species
PBMCs: Peripheral blood mononuclear cells
TCR: T cell receptor
NOX2: NADPH oxidase
O2^−^: Superoxide
H_2_O_2_: Hydrogen peroxide
ONOO^−^: Peroxynitrite
CMPs: Common myeloid progenitors
GMPs: Granulocyte–macrophage progenitors
GPs: Granulocyte progenitors
MPs: Monocytic progenitors
STAT3: Signal transducer and activator of transcription3
Treg: Regulatory T
TAMs: Tumor-associated macrophages
FOXP3: Forkhead box transcription factor
CAFs: Cancer-associated fibroblasts
IDO: Indoleamine oxidase
ATRA: All-trans retinoic acid
HNSCC: Head and neck squamous cell carcinoma
NSCLC: Non-small cell lung cancer
PDE5: Phosphodiesterase-5
ADC: Antibody–drug conjugate
HADCi: Histone deacetylase inhibitors
CTLA-4: Cytotoxic T-lymphocyte antigen-4
scRNA-seq: Single-cell RNA sequencing
